# Current Advances in 3D Bioprinting Technology and Its Applications for Tissue Engineering

**DOI:** 10.3390/polym12122958

**Published:** 2020-12-11

**Authors:** JunJie Yu, Su A Park, Wan Doo Kim, Taeho Ha, Yuan-Zhu Xin, JunHee Lee, Donghyun Lee

**Affiliations:** 1 Department of Biomedical Engineering, School of Integrative Engineering, Chung-Ang University, 221 Heukseok-Dong, Dongjak-Gu, Seoul 06974, Korea; junjie0801@hotmail.com; 2 Department of Nature-Inspired System and Application, Korea Institute of Machinery & Materials, 156 Gajeongbuk-Ro, Yuseong-Gu, Daejeon 34103, Korea; psa@kimm.re.kr (S.A.P.); wdkim@kimm.re.kr (W.D.K.); 3 Department of 3D Printing, Korea Institute of Machinery & Materials, 156 Gajeongbuk-Ro, Yuseong-Gu, Daejeon 34103, Korea; taehoha@kimm.re.kr; 4 Department of Engineering Mechanics, School of Mechanical and Aerospace Engineering, Jilin University, No. 5988, Renmin Street, Changchun 130025, China; xyz0208@jlu.edu.cn

**Keywords:** 3D bioprinter, natural polymer, synthetic polymer, bio-ink, tissue engineering

## Abstract

Three-dimensional (3D) bioprinting technology has emerged as a powerful biofabrication platform for tissue engineering because of its ability to engineer living cells and biomaterial-based 3D objects. Over the last few decades, droplet-based, extrusion-based, and laser-assisted bioprinters have been developed to fulfill certain requirements in terms of resolution, cell viability, cell density, etc. Simultaneously, various bio-inks based on natural–synthetic biomaterials have been developed and applied for successful tissue regeneration. To engineer more realistic artificial tissues/organs, mixtures of bio-inks with various recipes have also been developed. Taken together, this review describes the fundamental characteristics of the existing bioprinters and bio-inks that have been currently developed, followed by their advantages and disadvantages. Finally, various tissue engineering applications using 3D bioprinting are briefly introduced.

## 1. Introduction

The goal of tissue engineering, which is based on scaffold-based approaches, is the replacement/regeneration of damaged tissues or organs. A key prerequisite for such scaffold-based approaches is that the scaffold should be biodegraded after tissue restoration. Moreover, the structural design of scaffolds is important for successful tissue regeneration because the 3D structural characteristics and physical properties of native tissues/organs can play an essential role in the biological/physiological characteristics through appropriate cell–cell and cell-matrix interactions [1]. Additionally, scaffolds offer a suitable microenvironment for cell attachment, proliferation, and migration to promote cell growth and function [2].

Conventionally, scaffolds are fabricated using solvent casting/particulate leaching [3], gas foaming [4], melt molding [5], phase separation [6], freeze-drying [7], and electrospinning [8]. Although such manual methods allow the fabrication of porous structures, these processes could not construct tailored/regular porous structures. Notably, seeding cells on scaffolds cause considerable cell loss, resulting in poor cellular performance [9]. More important, it is difficult to create heterogeneous and multicellular structures mimicking actual tissues/organs using such conventional methods. Collectively, new biofabrication methods are necessary for advanced tissue engineering.

**Figure 1:**
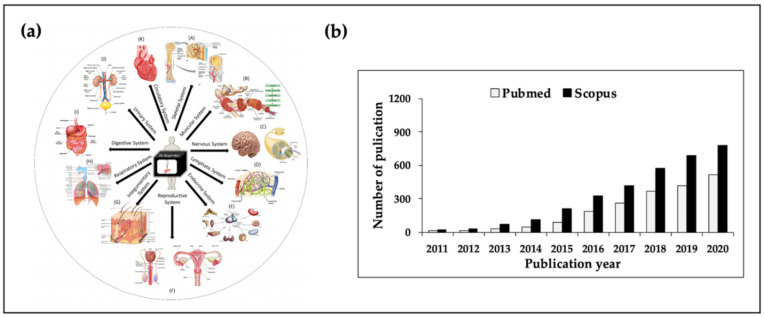
(**a**) Numerous applications of tissue engineering and (**b**) the number of publications based on 3D bioprinting. The image (**a**) was adapted with permission from [16]. Copyright 2018 Elsevier; illustration of the (**b**) was using search the terms of “3D bioprinting”. Data analysis was searched Pubmed and Scopus system on 11 November 2020.

3D bioprinting is an attractive biofabrication method because it enables the precise deposition of various cells/biomaterials onto predefined locations [10,11,12]. Additionally, it offers several advantages when compared with conventional methods. For example, this automated technique facilitates both mass production and high-throughput production with high-resolution [13]. Furthermore, customized structures for patients can be printed through computer-aided design modeling. Moreover, 3D bioprinted regular and porous structures can provide superior interconnectivity for cell growth/function. These advantages could lead to better tissue and organ regeneration (Figure 1a). Moreover, many outcomes have been published annually based on 3D bioprinting (Figure 1b).

Considering the working principles of tissue engineering, bioprinting techniques can be categorized into four modules: (1) droplet-based, (2) extrusion-based, (3) laser-assisted, and (4) stereolithography techniques. For the fabrication of an ideal 3D biomimetic structure using such printing modules, bio-inks should be carefully selected. To be used as a source of a bio-ink, biomaterials should meet the following requirements: (1) biocompatibility, (2) biodegradability, (3) bioprintability, and (4) structural integrity after printing [14,15]. To satisfy such requirements, various bio-inks have been formulated by considering their mechanical properties; in particular, mixtures of different biomaterials have attracted attention as potential bio-ink sources.

Over the past decade, research on the use of two or more bio-inks that combine cells has garnered great interest in the regeneration of artificial tissues. Various types of cells have been used in mixtures of different bio-inks to achieve remarkably successful results in the repair of bone, cartilage, trachea, blood vessels, and liver tissue. Although several studies related to 3D bioprinting have been reported, the objective of this review is to provide useful information regarding the current state of 3D bioprinting techniques and bio-inks. Moreover, based on each bio-ink characteristic, we propose blends of two or more bio-inks for use in various tissue engineering applications. Notably, in this review, we provide insights into the combination formulas of bio-inks, cell types, cell density, and crosslinkers, which are major challenges for building successful structures. Finally, the current limitations and future prospects are discussed.

## 2. 3D Bioprinters for Tissue Engineering

**Figure 2:**
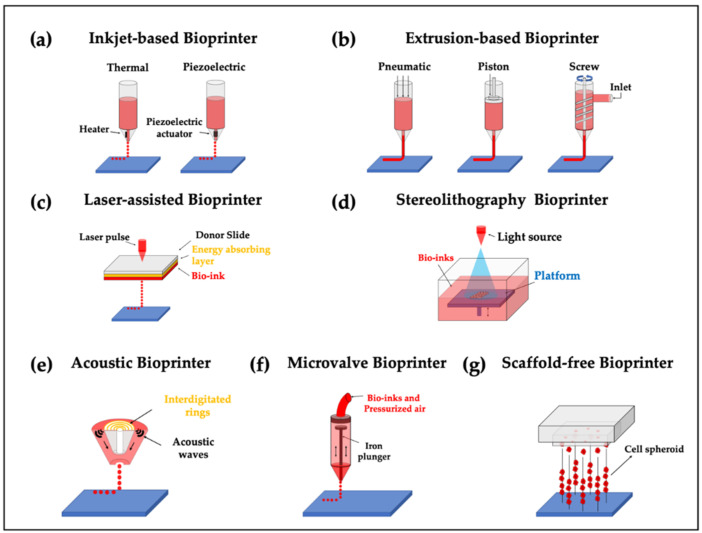
Different types of 3D bioprinters. (**a**) Inkjet- and (**b**) extrusion-based bioprinters were reproduced with permission from [17]; Copyright 2013 John Wiley and Sons. (**c**) laser-assisted bioprinter was reproduced from [18]; (**d**) stereolithography-based bioprinter was reproduced from [19]; (**e**) acoustic and (**f**) microvalve bioprinters were reproduced permission from [20]. Copyright 2016 Elsevier; (**g**) scaffold-free bioprinter was reproduced from [21].

As mentioned previously, 3D bioprinters can be commonly classified into four groups based on their working principles. In this section, we introduced seven types of bioprinters: (1) inkjet-based, (2) extrusion-based, (3) laser-assisted, (4) stereolithography, (5) acoustic, (6) microvalve, and (7) needle array bioprinters (Figure 2). We also provide a brief overview of the working principles of each printing module and its fundamental characteristics. The type of bioprinter should be carefully selected based on the structural properties of the targeted tissues/organs.

### 2.1. Inkjet-Based Bioprinters

Inkjet-based bioprinters were first reported in 1988 by Klebe; he utilized a commercially available Hewlett-Packard (HP) thermal drop-on-demand inkjet printer to print using a hydrogel solution [22]. Subsequently, inkjet-based printing modules have been successfully adopted to deposit cells or biomaterials as a droplet unit through various dispensing forces based on heating reservoirs or piezoelectric actuators. The heating element adjacent to the printing nozzle increases the temperature, which eventually causes gasification while generating bubbles [23,24]—the generated bubbles are forcefully printed as droplets on a substrate. In contrast, piezoelectric inkjet-based bioprinters generate pressure pulses that print cell-containing droplets through the nozzle [23]. Although inkjet-based bioprinters possess several advantages, such as high print speed and low cost [25], their application is limited because of the narrow ranges of printable biomaterial viscosities [17]. Heat-based and piezoelectric-based printing modules, owing to their working principles, may cause cell damage and cell lysis during the printing process [26]. However, the heating element only lasts a few microseconds at high temperatures; the cell viability of the printed cells can be maintained at 89%, with only a few cells being damaged using a thermal inkjet printer [27]. Moreover, non-uniform droplet size and nozzle clogging make the process cumbersome [25].

### 2.2. Extrusion-Based Bioprinter

Extrusion-based bioprinters were first introduced in 2002 [28]. Such printers deposit hydrogels through the forces exerted by pneumatic pressure or mechanical tools (piston or screw). When compared with inkjet-based bioprinters, extrusion-based printers can deal with high cell density, viscosities, and dynamical crosslinking mechanisms [29]. Additionally, extrusion provides a varied selection of biomaterials, including synthetic polymers, cell-laden hydrogels, cell aggregates, and microcarriers, because it enables the use of a wide range of biomaterial viscosities [29,30]. Moreover, they can produce a cell-laden bio-inks in the form of continuous extruded strands which is capable of engineering a large-scale biomimetic structure by use of their speedy printing velocity [10]. Despite these advantages, the relatively low resolution and poor cell viability due to the shear damage caused by the printing nozzle through pressure or mechanical force need to be ameliorated [31,32,33].

### 2.3. Laser-Assisted Bioprinter

Laser-assisted bioprinters were first introduced in 1999 by David Odde using optical cell trapping [34]. This system consists of an energy-absorbing layer, a donor ribbon, and a bio-ink layer [35]. In brief, a laser illuminates a small part of the donor ribbon layer, and a high-pressure bubble is created. The bubble pushes the bio-ink layer while generating droplets so that the bio-ink can be deposited on the substrate. During the printing process, the risk of contamination is low because the dispenser and the bio-inks are not in contact [36]. The main advantage of this system is that it can deposit bio-inks with relatively high viscosity and resolution [37]. Moreover, the issue of nozzle clogging is eliminated because this system involves a nozzle-free printing process [25]. In a previous study on laser-assisted bioprinters, Catros et al. conducted Ea.hy926 cell viability tests using a live/dead assay [38]. They reported that cell viability is related to the laser pulse energy, substrate thickness of the extracellular matrix (ECM), and viscosity of bio-inks. The results indicated that higher laser energy tends to increase cell damage. To prevent cell mortality, a higher thickness of substrate and viscosity could protect the cells in the bio-ink. Therefore, the potential inducement of cell damage due to laser intensity, high cost of printing modules, and difficulty in use are regarded as the main disadvantages of this technique [25,37].

### 2.4. Stereolithography Bioprinters

The first stereolithography was introduced by Charles W. Hull in 1986 [39]. Compared with the inkjet-based, extrusion-based, and laser-assisted bioprinting techniques, this method uses light to crosslink the bio-inks in the reservoir using a layer-by-layer process. Owing to its working mechanism, this technique is limited to light-responsive bio-inks, typically including gelatin methacrylamide (GelMa) and polyethylene glycol diacrylate (PEGDA) [40]. In addition to the limitations of options with bio-inks, another main disadvantage of stereolithography is that the reservoir may be filled with photopolymers, which entails material waste and a high cost of experimentation.

### 2.5. Additional Bioprinters

Acoustic and microvalve bioprinters are categorized as droplet-based bioprinters. An acoustic bioprinter ejects droplets when a force is generated using acoustic waves [41]. Compared with the abovementioned inkjet- and extrusion-based bioprinters, the living cells in bio-inks are not exposed to heat or high pressure that causes cell damage [20]. A microvalve bioprinter ejects droplets using an electromechanical microvalve consisting of a valve coil and plunger [20]. In brief, a magnetic field is generated by the valve coil, which forces the plunger upwards. Bio-inks in the barrel are pressurized by the resulting pneumatic pressure and are then ejected through the unblocked barrel. However, because the droplets generated from microvalve bioprinters are larger than those from inkjet-based bioprinters at the same nozzle size, the resolution is lower [20]. The Kenzan method is a scaffold-free method that laces pre-formed cell aggregates or spheroids onto a needle-array platform. LaBarge et al. developed a novel 3D bioprinter by fabricating entire layers of the construct at once, which alleviated the problem that only single spheroids were placed on the needle array at a time (i.e., one-by-one) [21].

### 2.6. Hybrid Printing Strategies

As noted previously, each 3D bioprinting module has its own inherent characteristics. To develop a highly complex/ideal tissue construct, further approaches based on combinations of such printing modules are required. Numerous efforts have been made to develop comprehensive structures using multiple printing modules, such as an inkjet-based bioprinter integrated with electrospinning [42], extrusion-based bioprinter combined with electrospinning [43], integrated inkjet-based and extrusion-based bioprinter [44], and laser-assisted bioprinter combined with electrospinning [45]. As a representative example, Xu et al. enhanced the mechanical strength of the cellular construct by combining an inkjet-based bioprinter with electrospinning for cartilage tissue engineering [42]. The supportive polycaprolactone (PCL) mesh was electrospun between the cellular layers to improve the mechanical strength of the structure. Kim et al. fabricated a composite scaffold using extrusion and electrospinning methods [43] and demonstrated that the composite structure exhibited enhanced mechanical and biological performance. Kim et al. suggested a hybrid bioprinting system that could simultaneously use inkjet-based and extrusion-based bioprinting modules to engineer 3D in vitro skin models [44]. Collectively, the hybrid printing strategies can produce structures with better mechanical and biological activities than conventional 3D bioprinting. However, this strategy requires more complex fabrication processes and more complicated software modes and hardware controllers, making it difficult for potential researchers to use such printing systems.

## 3. Bio-Inks: Biomaterials for 3D Bioprinting

**Figure 3:**
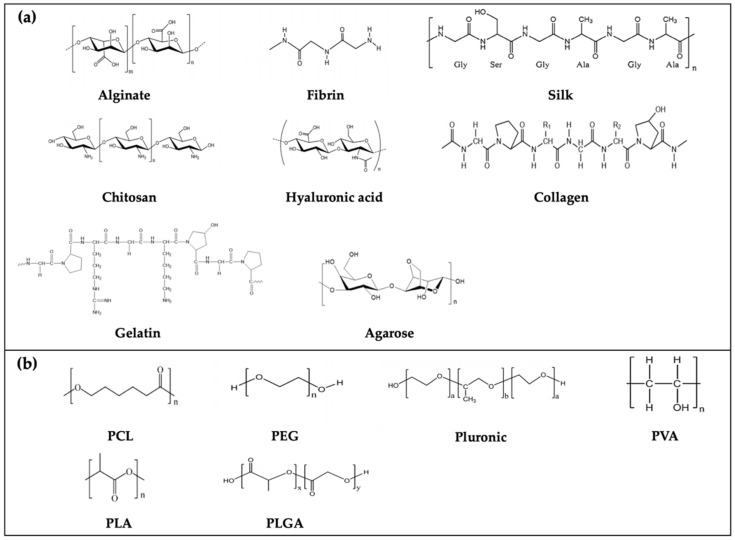
Chemical structure of (**a**) nature and (**b**) synthetic polymers. The chemical structure of alginate, fibrin, chitosan, hyaluronic acid, polyethylene glycol (PEG), polylactic-co-glycolic acid (PLGA) and pluronic were adapted with permission from [129]. Copyright 2017 Elsevier; silk and polyvinyl alcohol (PVA) were adapted with permission from [130]. Copyright 2017 Elsevier; polycaprolactone (PCL) and polylactic acid (PLA) [131]; agarose and gelatin [132]; collagen [133].

**Figure 4:**
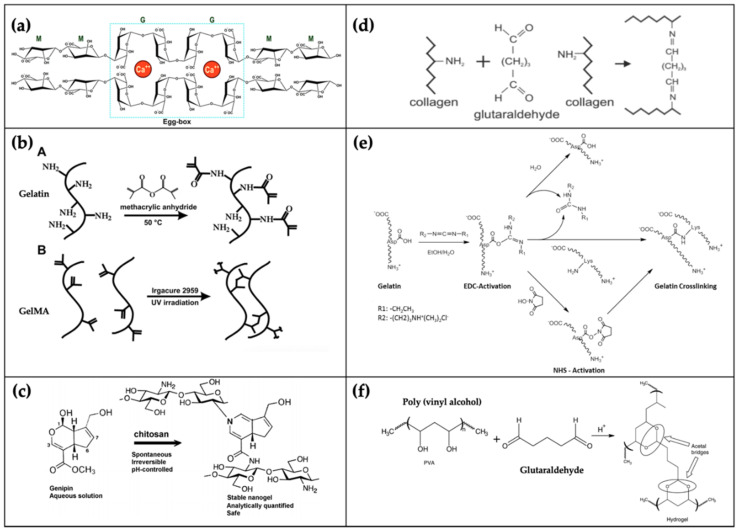
Crosslinking mechanisms of some instances. (**a**) alginate crosslinking with calcium ions (adapted from [134]); (**b**) synthesis gelatin methacrylamide (GelMA) and crosslinking with UV was adapted with permission from [65]. Copyright 2010 Elsevier; (**c**) chitosan crosslinking with genipin [135]; (**d**) collagen crosslinked with glutaraldehyde was adapted with permission from [136] Copyright 2018 Elsevier; (**e**) crosslinking mechanism of gelatin [137]; (**f**) PVA crosslinked with glutaraldehyde was adapted from [138]. Copyright 2008 Elsevier.

In parallel to the technological advances in 3D bioprinting, bio-inks (termed as printable hydrogels) are another key element for engineering functional tissue constructs. Biomaterials used for the manufacture of bio-inks should be biocompatible, bioprintable, and degradable in the human body without toxic byproducts. Here, we introduce conventional bio-inks based on natural and synthetic polymers and describe their features (Table 1). To better understand the chemical structure of the abovementioned polymers (Figure 3), some examples of crosslinking mechanisms (Figure 4) are provided. Finally, other bio-inks that have recently been adopted for 3D bioprinting are briefly covered.

### 3.1. Natural Polymers

Natural polymers, especially in the form of hydrogels, have the advantage of providing a favorable microenvironment for encapsulated cells. Here, we explain various types of natural polymers that are applied as bio-ink sources and their basic characteristics.

#### 3.1.1. Alginate

Alginate is a natural polymer derived from brown seaweeds [46]. Because its polymeric backbone is negatively charged, alginate can form ionically crosslinked chains by applying a solution with a positive charge. Calcium chloride (CaCl_2_) is well known as a typical solution that allows the alginate hydrogel to be ionically crosslinked [47]. Calcium sulfate (CaSO_4_) and calcium carbonate (CaCO_3_) can also be used as crosslinkers for alginate gelation; however, because their water solubility is inferior to that of CaCl_2_, the time required to crosslink the alginate increases accordingly [48].

Alginate-based hydrogels have been extensively employed for many biomedical applications owing to their biocompatibility, low toxicity, and relatively low price [47]. However, alginate hydrogels, based on ionic crosslinking, have limitations in terms of maintaining long-term stability under physiological conditions [47]. Another limitation of the use of alginate is that it does not provide binding sites for cell attachment [49]. This means that merely using alginate hydrogel would hinder cellular growth/function, and therefore, further approaches, such as the use of RGD peptide conjugation in combination with other cell-friendly biomaterials, should be implemented [47,50].

#### 3.1.2. Chitosan

Biocompatibility, biodegradability, nonallergenicity, and antimicrobial activity are the advantageous properties of chitosan, because of which it is widely employed in the engineering of various tissues, such as bone, cartilage, skin and liver [51,52]. The solubility of chitosan depends on the pH, and the bio-ink can be gelled at 40 °C under neutral conditions [23]. Genipin [53] and glutaraldehyde [54] can stabilize chitosan through a chemical crosslinking mechanism. The major disadvantages of chitosan are its weak mechanical integrity and rapid dissociation under certain physiological conditions, and the absence of cell-binding domains limits cell attachment [49]. To address these challenges, a chitosan-based blended bio-ink was developed by Ng et al., who formulated a mixture of polyelectrolyte gelatin and chitosan and optimized it for 3D bioprinting [55]. The results showed that the mixture maintained higher cell viability over four days of culture when compared with the pure chitosan group.

#### 3.1.3. Gelatin

Gelatin is a natural polymer obtained from animal connective tissues. It can be divided into two categories: (1) acid treatment of type A gelatin and (2) alkaline treatment of type B gelatin [49]. Different types of gelatin exhibit distinct characteristics—(1) type A and B show positive and negative charges at neutral pH [56] respectively; (2) type B shows a lower gel strength [57] but has better biocompatibility than type A [56], and (3) both are widely used in tissue engineering as bio-inks. Singh et al. mixed collagen type B and silk for the optimization and development of a crosslinker-free and printable bio-ink in cartilage engineering [58]. Erkoc et al. blended gelatin type A, cellulose, and alginate to conduct swelling and degradation tests using crosslinkers of glutaraldehyde or CaCl_2_ [59]. Based on these previous studies, the selection of gelatin type was dependent on the purpose of use. Moreover, it exhibits thermo-reversible gelation properties. Specifically, at low temperatures, it has a gel-like form, and at high temperatures, it can easily exhibit liquefied form. To induce irreversible gelation of gelatin hydrogels, glutaraldehyde can be used as a chemical crosslinker because of its high crosslinking efficiency [60]. However, glutaraldehyde is toxic to living cells. Therefore, alternative crosslinkers have been proposed, including transglutaminase, horseradish peroxidase (HRP) and H_2_O_2_ [61]. Recently, carbodiimide and genipin have been considered as potential candidate crosslinker for gelatin because their cytotoxicity is relatively lower than that of the other crosslinkers [62]. Although gelatin has biocompatible properties, non-immunogenicity and cell-friendly binding domains, pure gelatin cannot be used as a bio-ink source for cell growth because of its low viscosity and poor mechanical strength at 37 °C [23,49,63]. More recently, gelatin was modified with methacrylamide [64] and methacrylate [65] groups. Methacrylation makes the gelatin photocrosslinkable, with the developed gelatin being termed gelatin methacrylamide (GelMA). Many outcomes have been accumulated using this bio-ink [66,67,68]. As a representative example, Colosi et al. blended alginate/GelMA and bioengineered heterogeneous and functional 3D tissue structures [66].

#### 3.1.4. Collagen

Collagen is the main protein component of the ECM in actual tissues/organs [69], and sources of collagen have been obtained from animal tendon materials, such as rat and porcine [70,71]. Therefore, it has been extensively employed in the field of tissue engineering. Because of the rich integrin-binding domains, collagen provides superior microenvironments for cell growth, adhesion, and function [63,72]. Although collagen is in the form of a pre-gel at low temperatures, it can be thermally crosslinked when treated at 37 °C [36]. It can also be crosslinked with UV [73], glutaraldehyde [74], carbodiimide [75] and genipin [76] and is prone to degradation by collagenase [69]. However, it is difficult to print pure collagen because of its low viscosity. Therefore, several efforts are required to improve the viscosity of collagen; for example, the blends of collagen with other hydrogels [77,78] or the hybrid printing technique using synthetic polymers as a supporting framework to maintain the shape of printed collagen have been reported [79].

#### 3.1.5. Silk

Natural silk fibers that are produced by silkworms and spiders are an attractive source for the manufacture of bio-inks because of their nontoxicity, gradual degradation, and low immunogenicity [80,81]. Silk has a high viscosity and shear thinning inherently; these properties are advantageous for the fabrication of the desired structure [49]. The major disadvantage of silk is that nozzle clogging can easily occur owing to the shear force induced by β-sheet crystallization [82]. Moreover, the poor cell binding capacity of silk may limit cell adherence, growth, and function [49]. As a representative study, Das et al. reported sonication and enzyme-based crosslinking methods to improve the cellular function as well as the bioprintability of silk [83]. When a mixture of silk and gelatin was used as a bio-ink, mechanical integrity, cell viability, and differentiation were improved.

#### 3.1.6. Fibrinogen

Fibrinogen, which is essential for proper blood clot formation, can also be applied as a bio-ink source. It can be polymerized to fibrin through thrombin-fibrinogen interactions [84]. Fibrin has excellent biocompatibility, providing large binding sites for cell attachment and proliferation, as well as the inducement of minimal inflammation and low immunogenicity [85]. The main drawback of using fibrin as a bio-ink is that it is not suitable for a long time culture for in vivo applications because of its rapid degradation [63]. In addition, it is difficult to formulate fibrinogen itself as a printable hydrogel because of its low viscosity. Therefore, many studies using 3D bioprinting have used fibrinogen together with alginate, gelatin or collagen to improve its bioprintability during the printing process [78,86,87].

#### 3.1.7. Agarose

Agarose is a polysaccharide that is generally extracted from certain red seaweed [88]. Similar to other bio-inks, agarose is a hydrate and nonimmunogenic material, but it is brittle in a solid state [20,63]. However, its poor cell adhesion capacity [63] makes it unsuitable for use as a cell-laden biomaterial. It exhibits a sol–gel transition from 32 °C to 47 °C [89]. Due to its thermo-reversible feature, it normally works as a sacrificial bio-ink for hollow channels [90,91], rather than cell encapsulation and cell culture [77,92].

#### 3.1.8. Hyaluronic Acid

Hyaluronic acid (HA) is used because of its excellent biocompatibility and ability to form hydrogels with various chemical modifications [93,94]. However, HA hydrogels undergo rapid degradation and exhibit poor mechanical stability under physiological conditions [23]. To overcome these limitations, many researchers have attempted to blend HA with other suitable hydrogels [95,96].

#### 3.1.9. Matrigel

Matrigel is a gelatinous protein mixture derived from Engelbreth–Holm–Swarm (EHS) sarcoma cells and can be gelled at 37 °C [97]. It contains laminin, collagen, and a myriad of growth factors [98]. Matrigel is also a thermally reversible biomaterial and is in the liquid state at 4 °C; it exhibits a phase transition between 24 and 37 °C and requires approximately 30 min [97]. It has been reported that cells cultured on Matrigel exhibit exceptional cellular differentiation/functionalities when compared with those cultured on other homogeneous biomaterials [63]. Therefore, Matrigel may be a promising bio-ink source for successful tissue regeneration [63]. However, it has certain disadvantages. It is expensive and not suitable for clinical translation because of its origin [99]. Furthermore, Matrigel itself is not yet bioprintable. Therefore, it must be combined with other bio-inks to form a Matrigel-based bio-ink formulation [100,101].

#### 3.1.10. Bioceramics

Bioceramics are used as degradable and bioactive materials with excellent biocompatibility and antibacterial properties [102]; they include hydroxyapatite and a-, β-tricalcium phosphate. Owing to their restorability and bone conductivity, bioceramics have been widely used in bone healing applications [103]. Kim et al. combined bioceramics (β-tricalcium phosphate) with collagen to bioprint a 3D porous cell-laden structure [104]. They found that the addition of bioceramics with composite bio-ink significantly induced osteogenesis.

### 3.2. Synthetic Polymers

Synthetic polymer-based biomaterials are also a powerful source for manufacturing bio-inks because they can be precisely deposited with high fidelity and mechanical strength. However, poor biocompatibility and uncontrollable degradation remain a challenging issue. In this section, the fundamental properties of various synthetic polymer-based biomaterials, including PCL, polyethylene glycol (PEG), pluronic F-127 (PF127), polyvinyl alcohol (PVA), polylactic acid (PLA) and polylactic-co-glycolic acid (PLGA) are briefly covered.

#### 3.2.1. Polycaprolactone

PCL is a polyester-based biocompatible, and flexible material. It cannot encapsulate cells because melting or dissolving the polymer in organic solvents produces it in the liquid state, both of which are harmful to living cells [15]. Because of its relatively low melting point (~60 °C), high stability, and long-term degradation, PCL has been widely employed for 3D bioprinting-based tissue engineering [105]. In particular, PCL is used as a framework to support natural hydrogels with weak mechanical properties [106]. For example, Shim et al. developed a hybrid printing technique by depositing cell-laden natural hydrogels between the pores made of PCL [107]. With this technique, many beneficial results have been achieved in the engineering of tissue of the bone, cartilage, and liver [106,108,109,110,111,112].

#### 3.2.2. Polyethylene Glycol

PEG is a hydrophilic polymer that is widely used in 3D bioprinting because of its biocompatibility, non-immunogenicity, and protein rejection properties [113]. Owing to its high water solubility and hydrophilicity, this synthetic polymer is used as a sacrificial bio-ink [114]. To improve the mechanical strength, PEG can be modified using diacrylate (DA) [115] or methacrylate (MA) groups [116]. Bioprinted PEG-based systems can provide a 3D cell culture environment for various types of cells [117,118].

#### 3.2.3. Pluronic F-127

PF127 is a water-soluble and thermo-responsive material. At room temperature, it is in the gel state [15], but below 10 °C, it is in a liquid state [15]. It provides high fidelity for fabricating elaborate 3D structures. However, its mechanical integrity is too weak, and cells can barely grow in PF127 because of its poor cell support [49]. Therefore, PF127 has generally been used as a sacrificial bio-ink for generating perusable vascular structures [119].

#### 3.2.4. Polyvinyl Alcohol

PVA is a biodegradable, biocompatible, thermostable, and water-soluble synthetic polymer, which has already been approved by the Food and Drug Administration (FDA, USA) [120,121]. Glutaraldehyde, used as a cytotoxicity agent, can be crosslinked with PVA to obtain proper mechanical and physical properties [120]. However, because PVA exhibits poor cell affinity, its physical modification is achieved by alternative methods, such as freeze-thawing [120] and homogeneously blending with other hydrogels to obtain stable and intended composites [122,123].

#### 3.2.5. Polylactic Acid and Poly Lactic-co-Glycolic Acid

PLA and PLGA are polyester-based polymers, both of which are biodegradable, biocompatible, and have been approved by the FDA [124]. However, due to their inherent hydrophobicity, they have poor cell adhesion [124]. However, surface coating and plasma treatment can improve protein adsorption and enhance cell affinity [125]. Surface-modified bioprinted PLA and PLGA can provide a 3D cell culture environment for building various types of tissues [126,127,128].

### 3.3. Potential Candidates as Bio-Inks

The ECM supports tissue and arranges cells within connective tissues [139]. It can be harvested and decellularized from tissues such as bone, cartilage, and skin. The ECM contains numerous factors, including collagen, glycosaminoglycans, and elastin, which are advantageous for cell growth and differentiation [140]. Owing to the superiority of the ECM, decellularized ECM (dECM)-based bio-inks (also termed as tissue-specific bio-inks) have attracted attention. Decellularization processes that remove cellular components while leaving the ECM have been realized using various methods, including physical, chemical, and enzymatic treatments [141]. The existing dECM bio-inks are primarily thermally crosslinked because they are based on a collagen matrix [142]. However, it is difficult to build a 3D tissue construct with high shape fidelity owing to its low viscosity [49]. Therefore, various attempts have been made to overcome these restrictions by inducing rapid gelation or by co-printing synthetic polymers [142,143]. However, the critical limitations of using such tissue-specific bio-inks include batch-to-batch variation and complicated decellularization steps [15]. Furthermore, potential residues or toxic detergents after decellularization may impair cellular performance [144].

Recently, self-assembling peptides [145] and cellular aggregates/spheroids [146,147] have emerged as candidates for designing bio-inks. In particular, spheroids can augment cellular functions while promoting tissue formation [25]. Itoh et al. fabricated a 3D tube-shaped structure using multicellular spheroids and observed that tubular tissues were formed by remodeling and endothelialization in the abdominal aortae of nude rats [147]. Murata et al. also fabricated an osteochondral tissue by applying a scaffold-free method [148]. The pre-formed mesenchymal stem cell (MSC) spheroids were laced onto a needle array to form a columnar tissue using a 3D printer. The tissue morphology was transformed over six days of culture, and then the tissue was used as an autologous graft after extrication from the needle array. Although numerous tissue-mimicking constructs have been fabricated using spheroid fabrication techniques, necrosis may occur in the core of the tissue spheroid [63]. In addition, loading tissue spheroids into glass pipettes is still a challenging task because it causes nozzle clogging [63]. Deformation or breakage depends on their uniform size, maturation, and cell types [63].

Further, numerous nanobiomaterials have been incorporated into bio-inks for biomedical applications [149,150]. Furthermore, nanoparticle addition to polymeric hydrogels may result in the following physical and chemical modifications: (1) increase in stiffness, (2) shear-thinning, (3) controllable degradation, (4) enhancement of hydrogel networks with controlled drug release, and 5) photoresponsiveness [151,152]. Nanocellulose is one such nanoscale biomaterial—it can be divided into cellulose nanofibers and nanocrystals. The main source of cellulose is derived from plant cell walls and living organisms such as fungi, algae, and bacteria [153]. Nanocellulose integrates the unique features of cellulose, namely, high stiffness, modulus, hydrophilicity, and thermal stability [154,155] with the abovementioned properties of nanoparticles; consequently, nanocellulose composites are widely used in biomedical engineering combined with other bio-inks. Han et al. evaluated a composite of alginate/gelatin with the addition of different concentrations of nanocellulose for improving the printability of the composite [156].

Consequently, the introduced bio-inks based on natural and synthetic polymers, dECM, cell aggregates, spheroids, and nanocomposites have shown promising results for the development of functional tissues or organs using 3D bioprinting technology.

## 4. Current Applications of Tissue Engineering Based on 3D Bioprinting

Currently, there is a growing need for organ or tissue transplantation in tissue engineering because of donor shortage [157]. Multiple tissues have been successfully fabricated using 3D bioprinting, such as bone, cartilage, osteochondral tissue, blood vessels, liver, and organ-on-a-chip. To improve the printability and viability of bio-inks or to enhance the mechanical strength of the structure, complementary bio-inks have been developed in combination with two or more bio-inks. Here, we present tissue engineering applications based on 3D bioprinting. Additionally, we also listed the effective variables for successful biofabrication, including bio-inks, cell types, crosslinkers and 3D bioprinters in Table 2.

### 4.1. Bone Tissue

Bone is a hard tissue that supports the tissues and organs in the human body. Minor injuries of the bone tissue have self-healing capacity; however, major injuries require external stimulus for regeneration [158]. Until now, many outcomes have been accumulated for bone tissue engineering. Lee et al. reported a hybrid scaffold composed of PCL and cell-laden alginate [159]. They used PCL as a supportive framework to improve the mechanical strength of the construct. The results showed that the encapsulated cells in the alginate hydrogel were homogeneously distributed and exhibited approximately 84% cell viability, surviving well after 25 days of culture. In another study, Gao et al. evaluated osteogenic and chondrogenic effects when a mixture of PEGDMA–GelMA was used as the bio-ink [118]. The printed structure not only showed good cell viability (>80%) but also enhanced the degree of differentiation when compared with pure PEGDMA. Xavier et al. developed bioactive hydrogels composed of nanosilicates and GelMA [160]. The results showed that the viscosity of the composites increased at low shear rates, and the encapsulated preosteoblast cells were well grown without affecting cell viability relative to only GelMA hydrogel. Lee et al. bioprinted an hASC cell-laden mesh construct to evaluate the mechanical properties and cell viability using different formulas of hybrid bio-inks, which consisted of bone-derived methacrylated (Ma)-dECM and alginate [161]. The results showed that cell viability decreased after printing, owing to the higher viscosity, which occurred because of the increased concentration of Ma-dECM in the alginate. Furthermore, the addition of an appropriate concentration of Ma-dECM could promote cell proliferation and osteogenic differentiation. Zhang et al. provided a simple manufacturing method of hybrid bio-inks (alginate and gelatin) capable of bioprinting a porous bone-like tissue [162]. Different cell densities were used to observe cell viability and mineral deposition.

### 4.2. Cartilage

Cartilage is an avascular tissue that has a limited self-repair capability [163]. For cartilage tissue engineering, various strategies have been developed by formulating alginate-based hybrid bio-inks. To improve the resolution of the bioprinted structure, a nanofibrillated cellulose–alginate bio-ink was developed for cartilage tissue engineering [164]. The composite bio-ink exhibited high shape fidelity and resolution compared with pure alginate. It also provided high cell viability over seven days of culture. Kang et al. fabricated a human-scale ear using different types of bio-inks such as PCL, PF127, gelatin, fibrinogen, HA, and glycerol [165]. After printing the structure, PF-127 was liquefied, and the final ear construct was cultivated for further tissue maturation. Costantini et al. built a 3D biomimetic structure with either a mixture composed of GelMA and chondroitin sulfate aminoethyl methacrylate (CS-EMA) or GelMA, CS-EME and hyaluronic acid methacrylate (HAMA) by applying a coaxial dispensing technique [166]. To form a stable structure, alginate was used as a temporary agent. In the fabricated structure, the mixture composed of GelMA and CS-EME was observed to be the best substitute for cartilage formation. Ruiz-Cantu et al. developed a hybrid structure composed of PCL and chondrocyte-laden GelMA [167]. To manufacture an ideal structure, the temperature, needle gauge, crosslinking time, and different concentrations of GelMA were evaluated. Moreover, it was found that the addition of PCL to GelMA helped maintain the integrity of the porous structure, compared with the absence of the PCL groups. Recently, Ni et al. also fabricated hybrid bio-inks consisting of silk fibrin and hydroxypropyl methylcellulose [168]. Adding hydroxypropyl methylcellulose to the silk fibrin formed a double network capable of improving its mechanical strength. The results showed that the mechanical properties of the reported hybrid bio-inks were significantly improved compared with those of a single network.

### 4.3. Osteo-Cartilage

**Figure 5:**
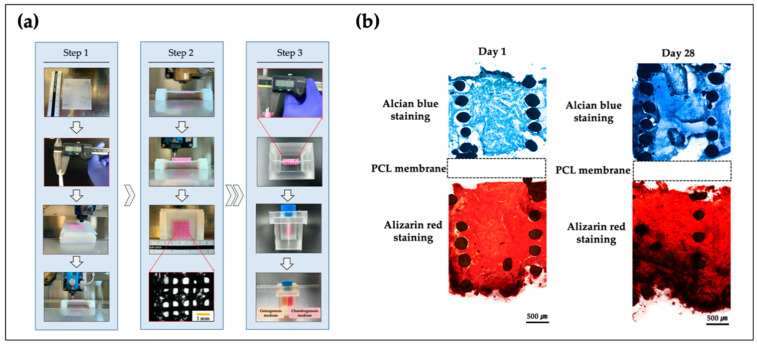
Fabrication process and histopathologic results of osteochondral scaffold [173]. (**a**) The fabrication process of bipartite scaffold using a 3D bioprinting system; (**b**) histological results at day 1 and 28.

To regenerate the osteochondral tissue composed of cartilage and subchondral bone, the two components should be simultaneously considered; however, fabricating such a heterogeneous structure has technical problems. In some studies, bone and cartilage were fabricated separately and assembled when they had matured [169,170]. However, this manual approach required a sophisticated process and thus lacked repeatability. As such, 3D bioprinting, which allows the use of various cells and biomaterials within a structure, fits well for osteochondral tissue engineering. Kosik-Kozioł et al. fabricated a triphasic scaffold consisting of noncalcified cartilage (made of an alginate solution reinforced with short PLA fibers), calcified cartilage (a hybrid scaffold composed of alginate, GelMA, and TCP), and subchondral bone (comprising a printed PCL porous structure modified with acetone and ultrasound) [171]. The triphasic scaffold was separately fabricated using three different types of bioprinters and subsequently binding their output using fibrin glue. Electrospinning is a typical conventional method; a combination of 3D bioprinting techniques has been used for the fabrication of controllable shapes of nano- and microscale constructs. Qiao et al. fabricated a triphasic scaffold combination of MSC-laden GelMA with the copolymer of PCL and PEG [172]. The copolymer scaffold was fabricated using the melt electrowriting method, which enhanced the mechanical properties of GelMA to maintain the entire structure. They found that the composite structure permitted growth factor loaded MSC, which was successfully differentiated with both cartilage and bone layers through in vivo tests. Yu et al. developed and characterized a heterogeneous scaffold using a multi-head 3D cell printing system [173]. The scaffold was achieved using PCL and alginate, allowing the embedment of a single type of progenitor cells that could be matured into two independent tissues simultaneously. They also developed a PDMS-based co-culture system to observe and evaluate for differentiation of osteochondral tissue (Figure 5). Overall, the findings suggested that these systems may find applications as an in vitro model for multilayered tissue formation.

### 4.4. Trachea

**Figure 6:**
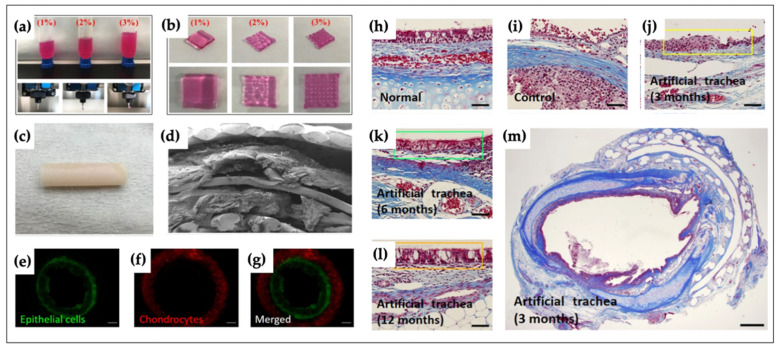
Characteristics of biofabricated artificial tracheal structure and histopathologic results of epithelial formation [174]. (**a**) 1, 3 and 5% alginate hydrogel being extruded through the ceramic nozzle; (**b**) optical image of alginate cube type; (**c**) optical image of biofabricated artificial trachea structure; (**d**) cross-sectional SEM image of bioprinted trachea; (**e**–**g**) cell tracker for epithelial cells (green), chondrocytes (red) and merged image; (**h**) normal tracheal epithelium; (**i**) control group; (**j**–**l**) experimental group at 3, 6 and 12 months (scale bar: 50 um); (**m**) a whole cross-sectional image of the experimental group at 3 months (scale bar: 1 mm).

The trachea, which has a cartilaginous tubular structure, transports air to the lungs. Various studies have attempted to regenerate/restore tracheal defects. For example, Park et al. engineered a tubular structure composed of PCL and alginate [174]. Autologous epithelial cells and chondrocytes were individually encapsulated in 3% alginate. Specifically, the trachea-mimicking structures included five independent layers. The first, third and fifth layers were composed of PCL, between which two bio-ink layers were printed. Afterward, the artificial trachea was transplanted into rabbits, and a respiratory epithelium was successfully formed (Figure 6). Ke et al. biofabricated a tracheal construct using PCL and cell-laden bio-inks [175]. The mechanical properties of the engineered tracheal structure were similar to those of native tissue. Recently, Kim et al. printed a two-layered hollow structure using electrospun 3D bioprinters [176]. The inner layer was made of nanoscale PCL fibers, and the outer layer consisted of microscale PCL fibers. After printing, different types of cells were loaded into the inner and outer layers to form a tracheal graft. Human bronchial epithelial cells were seeded into the inner layer, and cell-laden Matrigel (induced pluripotent stem cells derived mesenchymal stem cells/chondrocytes) was seeded on the surface of the outer layer to induce co-cultured cell attachment.

### 4.5. Skin

**Figure 7:**
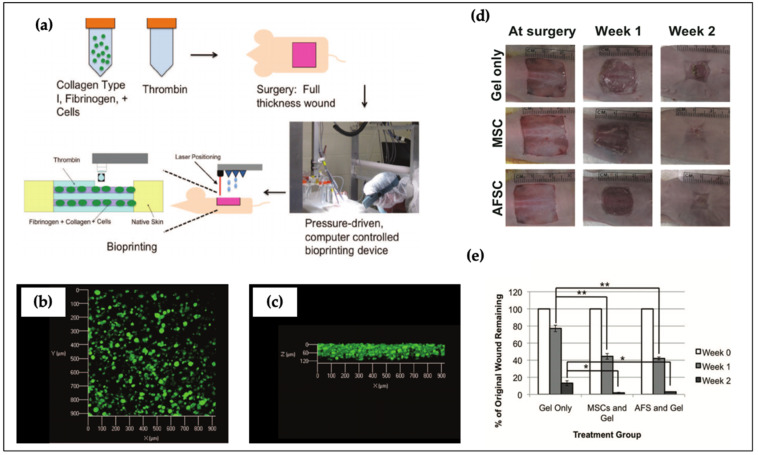
Biofabrication of artificial skin with hybrid bio-inks and assessment of wound closure by cell-free and cell treatment. (**a**) Schematic diagram of biofabrication process for wound closure; (**b**,**c**) cell distribution of top and side view after 24 h of culture; (**d**) histological results of wound closure and (**e**) wound remaining rate through the use of different treatments. Significance: *, *p* < 0.05; **, *p* < 0.01 Reproduced with permission from [78]. Copyright 2012 John & Wiley Sons.

The skin is composed of the epidermis, dermis, and hypodermis, which protects tissues and organs as a physical barrier. In the field of skin tissue engineering, several studies have reported that damaged skin tissue can be replaced by engineered artificial skin substitutes [177]. Skardal et al. developed a fibrin-collagen bio-ink and applied it for wound healing [78]. Specifically, human amniotic fluid-derived stem (AFS) cells and MSCs were separately encapsulated in bio-inks. The growth factors secreted by AFS cells promoted angiogenesis and wound closure. The results revealed that cells encapsulated within the bio-ink could significantly accelerate wound closure when compared with the cell-free group (Figure 7). Albanna et al. successfully bioprinted a 3D multicellular structure using fibroblasts and keratinocytes [178]. Cell localization and proliferation were evaluated for the formation of skin tissue. The printed construct exhibited rapid wound closure, and the regenerated region was considerably similar to healthy skin. Admane et al. proved that 3D bioprinted skin fabricated using a mixture of silk and gelatin was dimensionally stable relative to the collagen-based skin structure [179]. More recently, Hafezi et al. designed an alginate and chitosan–genipin–PEG-based three-layer skin tissue [180]. They also optimized the crosslinking ratio of chitosan and genipin to alter printability, comparable to that of commercial bio-ink. The results of cell viability showed that over 90% of cells lived after 24 and 48 h.

### 4.6. Neural Tissue

Neural tissue is similar to the abovementioned vascular networks. One study demonstrated that a novel 3D neural minitissue could be designed using a neural stem cell (NSC)-encapsulated bio-ink comprising alginate, agarose, and carboxymethyl-chitosan [92]. The results indicated that a uniform distribution of cellular constructs with high cell viability could be engineered using 3D bioprinting. Because NCSs can be differentiated into neurons and neuroglia, the neurons showed bicuculline-induced increased calcium response. In another study by England et al., Schwann cells encapsulated in a fibrin-HA-based bio-ink, which was printed in crosslinking solutions comprising PVA and thrombin, allowed the successful regeneration of nerve tissue [87]. The cells in the bio-ink remained viable and proliferated, and the fabricated fibrin fibers were longitudinally aligned. More recently, Liu et al. bioprinted a bi-layered nerve conduit with BMSCs and demonstrated that the structure has a great potential for the regeneration of peripheral nerve tissues [181]. The bi-layered nerve conduit comprised a GelMA-based inner layer for embedding BMSCs and a GelMA/PEGDA-based outer layer. The inner layer provided a cell growth environment for BMSCs, and the outer layer supported mechanical strength for the entire tubular structure. Next, PC12 cells were seeded into the inner layer, and it was found that attachment cells and the proliferation rate of PC12 cells were significantly higher than those for the absence of a BMSCs bi-layered nerve conduit. Li et al. formulated hybrid bio-inks consisting of alginate and Matrigel encapsulated with ectomesenchymal stem cells, which were used for neuron differentiation [182]. The results showed that cells induced growth and differentiation in bioengineered hybrid bio-inks. Wu et al. bioprinted a gelatin–alginate-based 3D construct to evaluate biocompatibility for in vitro/in vivo tests [183]. The results showed that over 90% of the Schwann cells survived after 24 h, and this was maintained over seven days of culture. Moreover, the secretion of a variety of neurotrophic factors by the Schwann cells, which were loaded into the 3D bioprinted construct, was significantly higher than that in the 2D culture.

### 4.7. Blood Vessel

**Figure 8:**
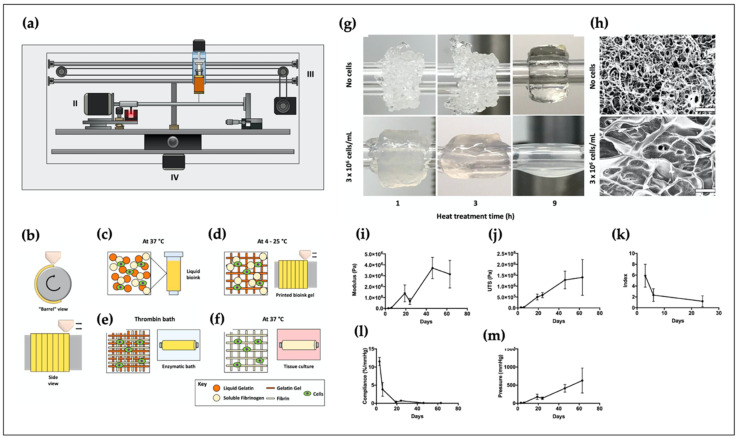
Development of a tubular structure using a rotary 3D bioprinting system. (**a**) A rotary 3D bioprinting system and (**b**-**f**) biofabrication process of a tubular construct in a whole research strategy; (**g**) optical image and (**h**) SEM images at the cell density of 1 × 10^6^ and 3 × 10^6^ cells/mL; circumferential (**i**) elastic modulus; (**j**) ultimate tensile strength (UTS); (**k**) anisotropy index; (**l**) compliance; (**m**) burst pressure of vascular constructs. Reproduced with permission from [187]. Copyright 2019 Elsevier.

Artificial blood vessels play an important role in connecting metabolically demanding organs, enabling the supply of nutrients while removing waste [184]. Jia et al. used alginate-based GelMA-4-arm poly(ethylene glycol)-tetra-acrylate (PEGTA) bio-ink for vascular tissue engineering [185]. The addition of PEGTA allowed tuning of the mechanical and rheological properties of the hollow construct. Gao et al. developed a bio-blood-vessel using a hybrid bio-ink containing alginate and vascular-derived dECM [186]. Endothelial progenitor cells (EPCs) and atorvastatin-loaded poly(lactic-co-glycolic) microspheres (APMS) were encapsulated in a bio-ink for the treatment of ischemic disease. A coaxially printed tubular structure was fabricated using the hybrid bio-ink and was allowed to evaluate its characteristics before implantation. In an in vivo test, neovascularization and significant regeneration were observed in the ischemic limbs of nude mice. Freeman et al. biofabricated small-diameter vascular constructs using a mixture of gelatin and fibrinogen [187] (Figure 8). In the hybrid bio-ink, gelatin was used to hold the printed fibrin during the printing process. They also observed that the collagen deposition, mechanical strength, and circumferential and axial elastic moduli increased after two months of culture. In Figure 8g, the addition of more cells into composites of gelatin-fibrinogen bio-ink impaired the gelation after heating treatment of the gelatin. Consequently, high cell density showed a more liquefied bio-ink in the work of Freeman et al. Recently, Jang et al. biofabricated a vascular scaffold (inner diameter: 4 mm, outer diameter: 5 mm, length: 40 mm) using two different bio-inks: alginate and PCL [188]. The artificial blood vessels included three independent layers. The first and third layers were composed of PCL, and 3% alginate was located between these two layers. Afterward, the bioprinted scaffold was transplanted into canines. Owing to the use of autologous MSCs, the cell-laden artificial structure was confirmed to have obtained better endothelialization with little inflammation.

### 4.8. Liver

**Figure 9:**
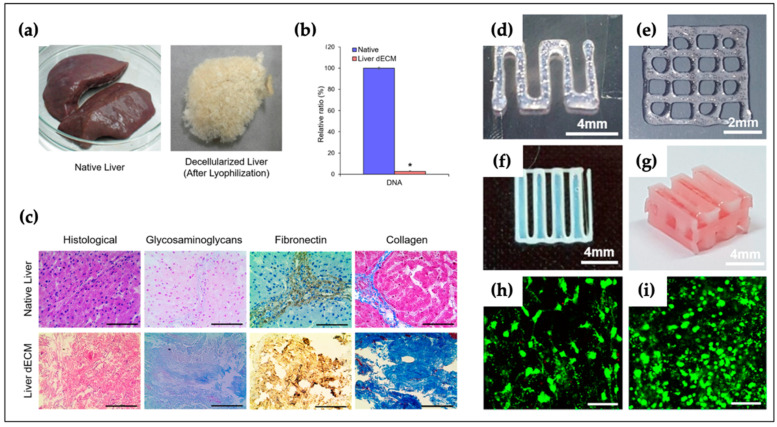
Development of liver-specific bio-ink for liver tissue engineering. (**a**–**c**) Evaluation of characteristics for liver-specific bio-ink relative to the native liver (*, *p* < 0.005; scale bar 100 um) (**d**) single line pattern and (**e**) 2D patterns; (**f**) 2D patterns using hybrid polymer and (**g**) 3D hybrid structures; (**h**) live/dead image of mesenchymal stem cell (MSC); and (**i**) HepG2 cell line. Adapted with permission from [189]. Copyright (2017) American Chemical Society.

The liver is dominated by hepatocytes and plays a key role in numerous metabolic activities. Lee et al. developed a hybrid construct consisting of a PCL and liver-specific dECM bio-ink [189]. Similarly, PCL was prepared as mechanical support to compensate for the weak mechanical properties of the dECM bio-ink. The dECM bio-ink was compared with a collagen type I bio-ink by focusing on the stem cell differentiation potency and human hepatocellular carcinoma (HepG2) cell function. Moreover, rich live cells were distributed homogeneously, and no red dots were observed in liver-specific dECM bio-ink (Figure 9). Wu et al. also bioprinted a liver-mimetic construct with a mixture of alginate and cellulose nanocrystals (CNCs) [190]. They demonstrated that a nozzle with an inner diameter of 100 µm could be utilized without clogging. Yang et al. bioprinted 3D liver functional tissues using HepaRG cells that combined gelatin and alginate composite bio-inks [191]. After verification of the 3D liver-like functional activity using the 3D bioprinted hepatoganoids, the construct was implanted into mice, and it was observed that the mice prolonged their survival in the experimental groups. Mao et al. bioengineered an inner gear-like construct using hybrid bio-inks consisting of GelMA and liver-derived dECM [192]. A liver-like structure was fabricated using a digital light processing bioprinter. The results showed that the cell viability, level of liver functional activities of albumin and blood urea nitrogen, and porosity were significantly higher than those in the absence of dECM. Gori et al. biofabricated a porous structure using composite bio-inks composed of PF127 and alginate [193]. They found printed 3D structures had better liver functional metabolism activity when comparing with the 2D cell adherent method.

### 4.9. Other Applications

Undoubtedly, 3D bioprinting can be used to develop a biological substitute that mimics the structural/physiological functions found in native tissues or organs. Beyond tissue regeneration, more recently, this technology has also been utilized for engineering in vitro tissue models and organ-on-chips. Homan et al. bioprinted perusable chips using human renal proximal tubules [194]. A silicone gasket was printed on a glass slide, and to form a tubular architecture; pluronic was used as the fugitive ink. A mixture of gelatin and fibrinogen was used as the ECM material, and thrombin was used as a crosslinker. The chips could relatively promote epithelium-like tissue formation compared with 2D controls. 3D bioprinting has also been applied for cancer modeling for drug screening. Dai et al. developed a 3D bioprinted brain tumor model, and the results of drug sensitivity tests demonstrated that the model exhibited enhanced endurance to temozolomide when compared with that of 2D monolayer cultivation [195].

## 5. Limitations and Future Perspectives

Considerable advances have been achieved in the field of 3D bioprinting with a large number of outcomes in terms of tissue engineering. However, this field is still in the early stages of development [37]. Therefore, a multidisciplinary collaboration will be an extremely important next step toward advanced tissue engineering. From the technological viewpoint, a higher printing speed for replicating clinically relevant sizes may be required.

The selection of bio-ink is another key factor for successful 3D bioprinting. Bio-inks should meet several rudimentary requirements, such as mechanical, rheological, and biological performances. Moreover, to maintain the entire structure over a long period of time, the printed structure requires appropriate stiffness; however, a very high stiffness can potentially impair cell viability [196]. Although many bio-inks have already been formulated and used in the field, efforts to manufacture new bio-inks should be continuously made to overcome the existing limitations. In particular, researchers developing new bio-inks should focus on balancing bioprintability and biofunctionality.

Apart from bio-inks, the cell source is another important point for successful tissue engineering. As such, a new method needs to be devised to accelerate cell expansion time without cell damage and mutations [20].

The development of microvasculature is important for maintaining the high cell viability of printed constructs over a long period of time. However, the fabrication of a similar native vascular network using current 3D bioprinting is limited because the size of the bioprinted tissues is larger than tens of micrometers.

Recently, 4D bioprinting technology has emerged as a powerful platform, which combines the “time value” with 3D bioprinting techniques. A 4D bioprinter can be constructed using a structure under different stimuli over time. The technique has the potential to engineer a more complex construct using stimuli-response biomaterials that can change the shape of the construct over time [197]. Therefore, shape memory polymers—called smart materials—have garnered attention in this field because they enable 4D bioprinting. Smart materials can recover their original shape via various external stimuli, including light, temperature, pH, and moisture [30]. Owing to the shape-changing ability that depends on time, printed structures using smart materials have been widely applied in biomedical areas [198,199,200]. Conclusively, 4D bioprinting may provide more favorable environments than conventional 3D bioprinting methods.

## 6. Summary and Conclusions

Obviously, 3D bioprinting has resulted in tremendous outcomes in tissue engineering. In this review, we first described representative bioprinters. Seven types of 3D bioprinting techniques were detailed, along with their advantages and disadvantages. Furthermore, various conventional bio-inks, including natural and synthetic polymers, dECM, and cell spheroids, were discussed. To improve the printability and cell viability, various combinations of bio-inks were applied for 3D bioprinting applications. We reported that the optimization of these bio-inks with proper 3D bioprinters would improve the probability of successful transplantation and regeneration of tissues/organs. Overall, we expect that this review will provide potential readers with beneficial and fundamental information on bioprinting technology and bio-inks for advanced tissue engineering in the future.

## Figures and Tables

**Table 1:** Current natural and synthetic bio-inks widely used for 3D bioprinting.

Bio-Ink	Crosslinking Mechanism	Advantages	Disadvantages	Ref.
Alginate	Ionic crosslinking	Biocompatibility, low toxicity, low price	Absence of cell-binding domains	[47,48,49]
Chitosan	Genipin, glutaraldehyde	Biocompatibility, biodegradability, antibacterial/fungal activity	Poor mechanical strength and rapid dissociation, absence of cell-binding domains	[49,51,52,53,54]
Gelatin	Temperature, glutaraldehyde, transglutaminase, HRP and H_2_O_2_, carbodiimide, genipin	Biocompatibility, non-immunogenicity and cell-friendly binding domains	Low viscosity and poor mechanical strength at 37 °C	[23,49,60,61,62,63]
Collagen	Temperature; UV, glutaraldehyde, carbodiimide and genipin	Improved cell adhesion, attachment, and growth	Low viscosity and poor mechanical strength	[36,63,72,73,74,75,76]
Silk	Enzymatic crosslinking	Nontoxicity, gradual degradation, and low immunogenicity; owning high viscosity and shear thinning	Inducement of nozzle clogging, absence of cell biding for cell adherence, limited cell growth and function	[49,80,81,82,83]
Fibrin		Cytocompatibility, providing binding sites for cell attachment, proliferation, and low immunogenicity	Rapid degradation, too soft, low mechanical strength and fragile	[63,85]
Agarose	Temperature	High mechanical strength, low price	Poor cell adhesion, brittle	[20,63,89]
HA	Glutaraldehyde, carbodiimide, divinyl sulfone	Enhancement of chondrocyte growth and chondrogenic differentiation	Rapid degradation and low mechanical strength	[23,93,94]
Matrigel	Temperature	Promotes cell growth and differentiation	Expensive and unsuitable for clinical translation	[63,97,98,99]
PCL		Low melting point and high stability	Unsuitable for cell encapsulation	[105]
PEG		Biocompatibility, non-immunogenicity; widely used sacrificial bio-ink	Low cell adhesion	[49,113,114]
GelMA	UV	Biocompatibility, biodegradable	Negative effects on cell viability in the crosslinking process	[49]
PF127	Temperature, UV	Commonly used as sacrificial bio-ink	Poor mechanical properties and unsuitable for cell culture	[15,49,119]
PVA	Glutaraldehyde	Biodegradable, biocompatible, thermostable, and water-soluble	Low cell affinity	[120,121].
PLA/PLGA		Biodegradable, biocompatible	Poor cell adhesion	[124,125]
dECM	Temperature	Promotes cell growth and differentiation	Low viscosity; complicated process of decellularization and costly; requires complete sterilization of dECM	[15,140,142,144]

**Table 2:** Recent works on composite bio-inks related to 3D printing.

Applications	Bio-Inks	Cell Types	Cell Density (Cells/mL)	Cross-Linkers	Types of 3D Bioprinters	Ref.
Bone	PCL/alginate	MC3T3-E1	2.3–2.8 × 10^5^	CaCl_2_	Extrusion	[159]
	PEG/GelMA	hMSCs	6 × 10^6^	UV	Inkjet	[118]
	GelMA/nanosilicates	MC3T3-E1	2 × 10^5^	UV	Extrusion	[160]
	(Ma)-dECM/alginate	hASC	5 × 10^6^	CaCl_2_/UV	Extrusion	[161]
Cartilage	alginate/nano fibrillated cellulose	Nasoseptal chondrocytes	15 × 10^6^	CaCl_2_	Microvalve	[164]
	alginate/GelMA/CS-ASMA/HAMA	BM-MSCs	>10^7^	CaCl_2_/UV	Extrusion	[166]
	PCL/GelMA	Chondrocytes	10^7^	UV	Extrusion	[167]
	silk fibroin/ hydroxypropyl methylcellulose	BM-MSCs	10^7^	UV	Extrusion	[168]
Osteo-cartilage	PCL/alginate	Progenitor cells	5 × 10^6^	CaCl_2_	Extrusion	[173]
Tracheal	PCL/alginate	Epithelial cells/chondrocytes	1 × 10^6^	CaCl2	Extrusion	[174]
	PCL/HA/gelatin/Heprasil/Gelin-S mix	MSCs	1.5 × 10^7^	Acrylate, alkyne, UV	Extrusion	[175]
Skin	collagen/fibrinogen	AFS/MSCs	1.66 × 10^7^	Thrombin	Droplet	[78]
	fibrinogen/collagen	Fibroblasts/ keratinocytes	3.75 × 10^6^/7.5 × 10^6^	Thrombin	Inkjet	[178]
	Silk/Gelatin	fibroblasts/ keratinocytes	2 × 10^6^/5 × 10^6^	Tyrosinase	Extrusion	[179]
Nerve	alginate/agarose/carboxymethyl-chitosan	Neural stem cell	1 × 10^7^	CaCl_2_	Extrusion	[92]
	fibrinogen/HA	Schwann cells	2 × 10^5^	Thrombin	Extrusion	[87]
	GelMA/PEGDA	BMSC	1 × 10^6^	UV	Extrusion	[181]
	alginate/Matrigel	EMSCs	1 × 10^5^	CaCl_2_	Extrusion	[182]
	gelatin-alginate	Schwann cells	2 × 10^6^	CaCl_2_	Extrusion	[183]
Blood vessel	alginate/GelMA/PEGTA	HUVEC/MSC	3 × 10^6^	CaCl_2_/UV	Extrusion	[185]
	alginate/dECM	EPCs	1 × 10^7^	CaCl_2_/temperature	Extrusion	[186]
	gelatin/fibrinogen	Human dermal fibroblast	1 or 3 × 10^6^	Thrombin/temperature	Extrusion	[187]
	PCL/alginate	MSC	1×10^6^	CaCl_2_	Extrusion	[188]
Liver	PCL/dECM	HepG2/MSC	5 × 10^6^	Temperature	Extrusion	[189]
	alginate/cellulose nanocrystals	Fibroblasts/hepatoma cells	1 × 10^6^	CaCl_2_	Extrusion	[190]
	gelatin/alginate	HepaRG	1 × 10^6^	CaCl_2_	Extrusion	[191]
	GelMA/dECM	hepatocytes	2.5–3.0 × 10^6^	UV	Digital light	[192]
	PF127/alginate	HepG2/C3A	2 × 10^6^	CaCl_2_	Extrusion	[193]
